# Nonmotor Symptoms in a Malaysian Parkinson's Disease Population

**DOI:** 10.1155/2014/472157

**Published:** 2014-04-01

**Authors:** Shahrul Azmin, Abdul Manaf Khairul Anuar, Hui Jan Tan, Wan Yahya Nafisah, Azman Ali Raymond, Othman Hanita, Shamsul Azhar Shah, Mohamed Ibrahim Norlinah

**Affiliations:** ^1^Department of Medicine, Universiti Kebangsaan Malaysia Medical Centre, Jalan Yaacob Latif, Cheras, 56000 Kuala Lumpur, Malaysia; ^2^Department of Pathology, Universiti Kebangsaan Malaysia Medical Centre, Jalan Yaacob Latif, Cheras, 56000 Kuala Lumpur, Malaysia; ^3^Department of Public Health, Universiti Kebangsaan Malaysia Medical Centre, Jalan Yaacob Latif, Cheras, 56000 Kuala Lumpur, Malaysia

## Abstract

*Background*. The nonmotor symptoms are important determinants of health and quality of life in Parkinson's disease but are not well recognized and addressed in clinical practice. This study was conducted to determine the prevalence of nonmotor symptoms and their impact on quality of life in patients with Parkinson's disease. *Methods*. This was a cross-sectional study among patients with idiopathic Parkinson's disease. Exclusion criteria were a Mini Mental State Examination score of <21/30. Prevalence of nonmotor symptoms was determined using the NMSQuest. The severity of nonmotor symptoms and the quality of life were assessed using validated disease-specific questionnaires (PDQ-39 and NMSS). *Results*. A total of 113 patients consisting of 60 males and 53 females were recruited. The median duration of illness was 5.0 (2.0–8.0) years. The prevalence rate of nonmotor symptoms in our cohort was 97.3%. The most common reported nonmotor symptom in our cohort was gastrointestinal (76.1%). We found that the severity of the nonmotor symptoms was associated with poorer quality of life scores (*r*_*s*_: 0.727, *P* < 0.001). *Conclusions*. Nonmotor symptoms were highly prevalent in our patients with Parkinson's disease and adversely affected the quality of life of our patients. In contrast to western studies, the most common nonmotor symptom is gastrointestinal. The possibility of an Asian diet playing a role in this observation requires further study.

## 1. Introduction

Although Parkinson's disease (PD) has traditionally been considered a motor system disorder, it is now recognized to be a complex disease with diverse clinical features that include nonmotor symptoms (NMS). In general, NMS are divided into 4 domains: neuropsychiatric, sleep, sensory, and autonomic [1].

It is estimated that NMS affects at least one-third of patients with PD [2, 3]. The NMS occur throughout the stages of PD, and some even predate the development of motor symptoms. Recent evidence suggests that NMS such as olfaction, REM sleep behavior disorder, fatigue, and depression may be markers of a preclinical stage of PD [4–6]. This can be a stepping-stone towards a premotor diagnosis of PD and may become important when a neuroprotective agent becomes available in the future.

NMS can exacerbate the general deterioration of health of PD sufferers, especially if left untreated [7]. In addition, NMS are easily missed and may go unreported during consultations, unless specifically asked by the physician [8]. This is confounded by the fact that both physicians and patients may be unaware of what exactly constitutes NMS. In response, several questionnaires have been developed to address these problems and include the Non Motor Symptoms Questionnaire (NMSQuest) and Non Motor Symptoms Scale (NMSS), both of which have been extensively used and validated in patients with PD.

NMS are important determinants of quality of life (QoL). NMS such as depression and cognitive impairment can adversely affect the QoL of patients with PD [9]. Furthermore, poor QoL has been shown to be a predictor of institutionalization [10].

To expand and improve the understanding of NMS in PD, we used a battery of questionnaires to study the demographic characteristics of NMS in a cohort of consecutive patients with PD. We aimed to determine the prevalence of NMS in PD and how this affects the QoL of the patients.

## 2. Methodology

This was a cross-sectional observational study conducted at Universiti Kebangsaan Malaysia Medical Centre from December 2009 till August 2010. Consecutive PD patients aged 18 and older, attending the neurology clinic, were invited to participate in the study. Diagnosis of PD was made by a neurologist with competence in movement disorders according to the UK PD Brain Bank Criteria. Patients were excluded if they had a MMSE score of less than 21/30. The study complies with the Declaration of Helsinki and was approved by the institutional ethics committee.

Patients who agreed to participate and gave informed consent were interviewed and examined clinically. A semistructured interview was used to obtain information on disease history and other sociodemographic information such as gender, age, disease onset, and disease duration. The assessment included Hoehn and Yahr staging. Three sets of questionnaires were administered: Non Motor Symptoms Questionnaire (NMSQuest), Non Motor Symptoms Scale (NMSS), and Parkinson's Disease Questionnaire-39 (PDQ-39). Either the English or the Malay version of each of the questionnaires was administered according to the patients' language preference. The questionnaires were administered by a single interviewer.

### 2.1. Instruments

NMSQuest is a questionnaire comprising a series of 30 questions which helps to screen for the presence of NMS [11]. The instrument is a screening tool and does not give out a score of disability and is not intended to be used as a rating scale. NMSS is another questionnaire that has been developed to help evaluate the severity of individual nonmotor symptoms [12]. The frequency and severity of each NMS are taken into account and are reflected in the final total score of disability (0 = no disability; 360 = maximal disability). We used PDQ-39 questionnaire to measure health-related QoL. It is a disease specific self-reporting assessment tool for health status in patients with Parkinson's disease and has been validated extensively [13–15]. PDQ-39 questionnaire consists of 39 questions measuring eight dimensions of health: mobility, activities of daily living, emotional well-being, stigma, social support, cognition, communication, and bodily discomfort. Lower scores indicate better perceived health (0 = perfect health, 100 = worst health).

All data were analysed using SPSS 18.0 statistical software package. Qualitative and quantitative demographic characteristics were tabulated and tested for normality using* Shapiro-Wilk* test. Results were expressed by mean ± standard deviation (SD) or median with interquartile range (IQR).

To compare means of two normally distributed data, Student's *t*-test was used. For non-normally distributed data, Mann-Whitney *U*-test was used to compare between two groups. For comparison of proportions between two groups, chi-square and Fisher's exact test were used. Correlations between these variables were also assessed by the Spearman's Rho test.

A *P* value of <0.05 was deemed as statistically significant.

## 3. Results

A total of 113 patients who fulfilled the inclusion and exclusion criteria were enrolled into the study (Table 1).

The overall mean age of this study population was 64.8 ± 9.0 years. The mean age of onset of PD was 59.0 ± 9.9 years and the median duration of illness was 5.0 (2.0–8.0) years.

Seventy-one (62.8%) patients were Chinese, 32 (28.3%) were Malay, 8 (7.1%) were Indian, and 2 (1.8%) were from other races listed above. There were 60 (53.1%) males and 53 (46.9%) females in the study cohort.

The median PDQ-39 score was 13.0 (5.0–28.0) and the median Hoehn and Yahr stage was 2.0 (2.0–3.0).

### 3.1. Nonmotor Symptoms

Of the total 113 patients, 110 (97.3%) had NMS based on the NMSQuest. Gastrointestinal symptoms were the commonest NMS domain in our cohort and were reported by 86 patients (76.1%). The 2 most commonly reported gastrointestinal symptoms in our cohort were constipation and dribbling of saliva during daytime: 70 patients (61.9%) and 32 patients (28.3%), respectively.

The second most reported NMS domain in our cohort was neuropsychiatric symptoms, which was present in 82 patients (72.6%). Sixty-six patients (58.4%) complained of attention/memory problems, 60 patients (58.4%) complained of mood/cognition problems, and 15 patients (13.3%) complained of problems relating to perception/hallucinations (Table 2).

Autonomic dysfunction was present in 64.6% of patients, with urinary dysfunction being the most commonly reported sub-item in this domain (Table 2).

Olfactory dysfunction was reported in 21 patients (18.6%) and was the least frequent reported NMS domain in our cohort (Table 2).

### 3.2. Nonmotor Symptoms and Quality of Life

Among individual PDQ-39 dimensions, our study population had the lowest scores (i.e., better QoL) in the stigma dimension (median 0.0; IQR: 0.0-0.0). Our cohort recorded the highest scores (poorest QoL) in the cognition dimension (median 12.5; IQR: 6.3–18.8), followed by the emotional well-being dimension (median 8.3; IQR: 0.0–25.0) (Table 3).

### 3.3. Correlation of Nonmotor Symptoms Severity with Quality of Life

The severity of NMS in our study population was assessed using the NMSS that consists of 9 domains. The median scores for the individual NMSS domains of our study population are presented in Table 4.

We assessed whether there were any correlations between the severity of individual NMSS domains with the quality of life of our study cohort (using PDQ-39 scores). Except for domains assessing the sexual and urinary symptoms, the median NMSS score for all the other domains showed significant positive correlation with the total PDQ-39 scores (Table 4).

## 4. Discussion

This is the first study on NMS performed in the Malaysian PD population. There was a high prevalence of NMS among our PD patients. Out of the 113 patients studied, we found that 110 (97.3%) had at least one NMS. Others studies using the same assessment tools quote a NMS prevalence rate of at least 98% [16, 17]. Li et al. found an NMS prevalence rate of 100% in a cohort of 82 patients recruited at a tertiary hospital in mainland China [18]. It was suggested that the high prevalence rate was due to the lack of medical facilities and expertise, causing the patients to be referred late and at a stage where the symptoms had worsened and become more florid. Our study setting was similar to that of Li et al., but our findings were more comparable to those done in the Western population. This may be explained by the fact that the catchment area where our study was performed is serviced by 3 tertiary care centers with expertise in movement disorders, where patients are referred and diagnosed early in their disease.

We found that the most common reported NMS in our cohort were gastrointestinal symptoms. Gastrointestinal symptoms have been noted since the first description of PD in 1817 and is the most studied component of the NMS in PD complex [19, 20]. The pathophysiological process occurs at several levels across the central nervous system and the gastrointestinal tract. The dorsal motor nucleus of the vagus nerve which controls the parasympathetic nerve innervations of the gastrointestinal system is affected in PD [21, 22]. Dystonia of the striated external anal sphincter and the reduction of dopamine containing neurons within the colonic part of the enteric nervous system also contribute towards the development of gastrointestinal symptoms in PD [23–25]. It has been suggested that alpha-synuclein deposition within the enteric nervous system may be the earliest site of pathology of PD, thus signifying that possibly the gastrointestinal system is the portal of entry for the pathophysiological process that ultimately culminates in the clinical syndrome of PD [26]. Extrinsic factors also play a role in the development of gastrointestinal manifestations and this includes* Helicobacter pylori* infection of the duodenum and stomach. Nafisah et al. showed that the Malaysian PD population has an increased risk of having* Helicobacter pylori* infection and this may adversely impact on the motor and nonmotor symptoms of PD [27].

In our cohort, in those who complained of gastrointestinal symptoms, 70 patients (61.9%) specifically complained of constipation. Older studies investigating constipation in PD quote a lower prevalence rate of about 30% [28]. This may be attributable to a difference in definition and methodology. Nevertheless, the PRIAMO study conducted in an Italian population using the same assessment tools as ours quoted a constipation prevalence rate of only 27.5% [16]. A literature review looking at recent studies of NMS in PD using NMSQuest showed that the Asian PD population had a tendency to report a higher rate of prevalence of gastrointestinal symptoms, especially constipation, as compared to studies performed in the West (Table 5).

Regional variation in diet may contribute to the difference in the prevalence rate of constipation in PD patients in different parts of the world. It has been shown that diet rich in insoluble fibre has a beneficial effect on motor and nonmotor symptoms of PD [29]. A cross-sectional study of 117 PD subjects in China showed that poor nutrition was associated with several nonmotor symptoms, including constipation [30]. Asian countries in general are developing economies and the rate of poverty is higher than that in western countries. We are unable to determine how much this factor impacts on our findings, but nevertheless, it may partly explain the high prevalence rate of constipation found in our study and others performed in Asia. Finally, our finding may be cultural. The NMSQuest is a screening tool that is in the form of a self-administered questionnaire. The rate of positive responses for gastrointestinal symptoms may be coloured by the patients' outlook and philosophy towards their disease. It has been shown that the NMSQuest is better in detecting more disabling NMS, as it is perceived by the patient [31]. Our finding may suggest that the Asian PD patients consider gastrointestinal symptoms to be much more disabling and debilitating to their daily activities as compared to other symptoms such as anxiety, depression, and insomnia.

The second most common NMS domain in our cohort was neuropsychiatric symptoms (72.6%), followed by autonomic dysfunction (64.6%) and sleep disorders (58.4%). Our finding appears to be consistent with the PRIAMO study which found that 66.8% and 64.1% of their subjects complained of psychiatric and sleep disorders, respectively [16]. A study of NMS in PD in the Chinese population using NMSQuest also reported a high prevalence rate of neuropsychiatric symptoms and sleep disorders in their cohort [32].

Autonomic dysfunction is a common NMS in PD and includes urinary dysfunction. In general, the prevalence rate of urinary dysfunction amongst PD patients is quoted to range from 27% to 39% [33, 34]. Winge et al. using SPECT imaging demonstrated that the severity of urinary dysfunction correlated with the relative degeneration of the dopamine dependant caudate nucleus [35]. In our study, only 32.7% of patients complained of urinary symptoms. In contrast, Martinez-Martin et al. found that urinary symptoms featured prominently in their cohort with 59% of their patients complaining of some sort urinary dysfunction [17]. A possible explanation for the differing prevalence rate of urinary symptoms between our cohort and the cohort of Martinez-Martin et al. is the mean duration of PD, which is 5 years and 8 years, respectively. Interestingly, the PRIAMO study, which also had a cohort with a mean duration of illness of PD of 5 years, reported a prevalence rate of urinary symptoms of 35%, which is similar to our results [16].

In our study, we found that the NMS domain with the lowest prevalence rate was olfactory dysfunction, with only 18.6% of our patients answering positively on the NMSQuest. The prevalence rate of olfactory dysfunction in PD patients has been reported to range from as low as 45% to as high as 90% [36, 37]. The very wide difference in prevalence rate may be attributed to different methods in testing for olfactory dysfunction [38]. There are several reasons that may explain the low positivity rate of olfactory dysfunction in our study. Patients may fail to recognize that olfactory dysfunction is a problem, or if they do, they do not necessarily attribute it to Parkinson's disease [39, 40]. This is especially true if the olfactory dysfunction has developed before the more recognizable motor symptoms that people tend to associate with Parkinson's disease. The NMSQuest has been shown to have poor sensitivity in certain nonmotor symptoms, particularly olfaction, especially if the symptoms are mild and not particularly disabling [31]. This argument may also be the reason why only 30.1% of our cohort complained of sensory symptoms on NMSQuest.

### 4.1. Nonmotor Symptoms and Quality of Life

In our cohort, the “stigma” dimension (median 0.0; IQR: 0.0-0.0) followed by “social support” dimension (median 0.0; IQR 0.0–8.3) had the lowest score (better QOL) on PDQ-39 questionnaire. This suggests that these domains have the least adverse impact on the quality of life of our patients. We hesitate to put too much weight in this observation as it may just be a reflection of the demographic characteristics of our study population who are predominantly at an earlier stage of disease (48.7% at Hoehn and Yahr stage 2).

The NMSS domains of mood and cognition had the highest concordance with PDQ-39 scores. This is unsurprising as numerous studies have shown the adverse impact of depression and dementia on the QoL of patients with PD and our findings reinforce this fact. Depression has been shown to be the most important predictive factor of poor QoL in patients with PD [41, 42].

In contrast to our findings, Gallagher et al. using a different clinimetric tool found that the PDQ-39 had significant correlation with sexual function and this correlation is even more pronounced in patients with PD duration of less than 5 years [41]. Our finding may be cultural, as Asian tends to be more reserved and may not be as forthcoming when posed with questions relating to their sexual function. Indeed, Chaudhari et al. in the pilot study of NMSQuest also found that questions relating to sex were most frequently left unanswered [11].

Our study has some strength. We used standardized assessment tools that have been extensively validated in previous multicentre trials. To reduce interoperator bias, all questionnaires were administered by a single interviewer in a face-to-face interview. Our study also looked at the whole spectrum of NMS which provides a robust overview of the overall NMS experienced and its impact on QoL.

Our study is limited by the fact that we did not differentiate whether the patient was in an “On” or “Off” state during the interview. However, since this was a prevalence study, we felt that it was unnecessary to ascertain their “On” or “Off” state. Ideally, a case control study would have been better in giving information on the true prevalence of some of the commoner NMS, which are typically encountered in the elderly population.

## 5. Conclusion

The prevalence rate of NMS in our study was 97.3%. We found that gastrointestinal symptoms were the most prevalent NMS in our cohort. The role of regional diet variation in the development of gastrointestinal symptoms in PD patients should be explored further. Disorders of mood and cognition had the most adverse impact on the QoL than any other NMS. Our study reaffirms the view that it is important to recognize and treat the symptoms of depression early.

## Figures and Tables

**Table 1:** Baseline characteristics of study population.

	*n* = 118
Age; mean (SD)	64.8 (9.0)
Sex	
Male	60 (53.1%)
Female	53 (46.9%)
Race	
Malay	32 (28.3%)
Chinese	71 (62.8%)
Indian	8 (7.1%)
Others	2 (1.8%)
Age of onset of PD, years; mean (SD)	59 (9.9)
Duration of illness of PD, years	5 (2.0–8.0)
Hoehn and Yahr	2 (2.0–3.0)
Stage 1	26 (23%)
Stage 2	54 (48.7%)
Stage 3	30 (26.5%)
Stage 4	2 (1.8%)
Total PDQ-39 score	13.0 (5.0–28.0)
Total NMSS score	9.0 (6.0–15.0)

Data is expressed as median (IQR) unless otherwise stated.

PDQ-39: the 39-item Parkinson's disease questionnaire (range of score 0–100); NMSS: nonmotor symptoms scale for Parkinson's disease (range of possible scores 0–360).

**Table 2:** Frequency of NMS among the study population.

	*n* = 118; *n* (%)
Neuropsychiatric symptoms	**82 (72.6)**
Mood/cognition	60 (53.1)
Perceptual problems/hallucinations	15 (13.3)
Attention/memory	66 (58.4)
Sleep disorders	**66 (58.4)**
Autonomic dysfunctions	**73 (64.6)**
Urinary dysfunction	37 (32.7)
Sexual dysfunction	9 (8.0)
Orthostatic hypotension	50 (44.2)
Excessive sweating	14 (12.4)
Gastrointestinal symptoms	**86 (76.1)**
Olfactory dysfunction	**21 (18.6)**
Sensory symptoms	**34 (30.1)**
Miscellaneous	**50 (44.2)**

**Table 3:** Total scores of PDQ-39 dimensions.

PDQ-39 dimensions	Score
Mobility	5.0 (0.02–42.5)
ADL	0.0 (0.0–12.5)
Emotional well-being	8.3 (0.0–25.0)
Stigma	0.0 (0.0-0.0)
Social support	0.0 (0.0–8.3)
Cognition	12.5 (6.3–18.8)
Communication	0.0 (0.0–16.7)
Bodily discomfort	8.3 (0.0–16.7)
PDQ-39 total	8.5 (3.4–5.1)

Data is expressed as median (IQR).

PDQ-39: the 39-item Parkinson's disease questionnaire (range of score 0–100).

**Table 4:** Correlation between NMSS domains and PDQ-39 scores.

NMSS domains	Median (IQR)	*r*_*s*_	*P*
Cardiovascular	0.0 (0.0–2.0)	0.563	<0.001
Sleep/fatigue	2.0 (0.0–3.0)	0.469	<0.001
Mood/cognition	1.0 (0.0–3.0)	0.588	<0.001
Perceptual problems	0.0 (0.0-0.0)	0.336	<0.001
Attention/memory	1.0 (0.0–3.0)	0.281	0.003
Gastrointestinal	2.0 (0.0–3.0)	0.412	<0.001
Urinary	0.0 (0.0–1.0)	0.154	0.104
Sexual function	0.0 (0.0-0.0)	0.173	0.067
Miscellaneous	1.0 (0.0–2.0)	0.258	0.006
NMSS total	9.0 (6.0–15.0)	0.727	<0.001

*r*_*s*_: Spearman's rank correlation coefficient; NMSS: nonmotor symptoms scale for Parkinson's disease; PDQ-39: the 39-item Parkinson's disease questionnaire.

**Table 5:** Prevalence rate of gastrointestinal symptoms in studies using NMSQuest.

Source	*n*	Location	Prevalence
Khedr et al. [43]	112	Assiut, Egypt	76.80%
Gan et al. [44]	155	Shanghai, China	64.56% (constipation)
Jia et al. [45]	170	Nanjing, China	68.2% (constipation)
Bostantjopoulou et al. [46]	166	Thessaloniki, Greece	45.7% (constipation)
Tsuboi et al. [47]	53	Fukuoka, Japan	78.4% (constipation)
Cheon et al. [48]	74	Busan, Republic of Korea	65.8% (constipation)
Yu et al. [49]	90	Shandong, China	70% (constipation)
Gallagher et al. [41]	89	London, UK	48% (constipation)
Crosiers et al. [50]	215	Antwerp, Belgium	38.6% (constipation)
Cosentino et al. [51]	300	Lima, Peru	55.7% (constipation)
Rodríguez-Violante et al. [52]	100	Mexico City, Mexico	30%
Barone et al. [16]	1072	Italy	27.5% (constipation)
